# Transient Neonatal Diabetes: An Etiologic Clue for the Adult Diabetologist

**DOI:** 10.1016/j.jcjd.2019.05.002

**Published:** 2020-03

**Authors:** Anela Novak, Pamela Bowman, Ivana Kraljevic, Marija Tripolski, Jayne A.L. Houghton, Elisa De Franco, Maggie H. Shepherd, Veselin Skrabic, Kashyap A. Patel

**Affiliations:** a Section of Endocrinology, Department of Internal Medicine, University Hospital Split, Split, Croatia; b The Institute of Biomedical and Clinical Science, University of Exeter Medical School, Exeter, United Kingdom; c Department of Endocrinology, Department of Internal Medicine, University Hospital Zagreb, Zagreb, Croatia; d Section of Endocrinology, Department of Internal Medicine, University Hospital Osijek, Osijek, Croatia; e Exeter NIHR Clinical Research Facility, Royal Devon & Exeter NHS Foundation Trust, Exeter, United Kingdom; f Section of Neuroendocrinology, Department of Pediatrics, University Hospital Split, Split, Croatia

**Key Messages**

• Relapse of transient neonatal diabetes mellitus (TNDM), when it occurs in adulthood, presents a diagnostic challenge; affected individuals are often misclassified resulting in suboptimal treatment.
• Our report highlights the importance of TNDM in the medical history as a crucial etiological clue to the adult diabetologist.
• This allows diagnosis of monogenic diabetes, leading to appropriate treatment and genetic counselling.

## Introduction

Neonatal diabetes mellitus is a rare form of monogenic diabetes diagnosed before 6 months of age which occurs in approximately 1 in 100,000 live births (1). Nearly one-half of neonatal diabetes is transient, with remission within 1 to 18 months followed by relapse later in childhood or in adult life 2, 3. Almost 90% of cases of transient neonatal diabetes mellitus (TNDM) are caused by mutations in the ATP-sensitive potassium (K_ATP_) channel genes *KCNJ11* and *ABCC8* (4) or chromosome 6q24 imprinting abnormalities (5). Activating mutations in K_ATP_ channels, formed by Kir6.2 (*KCNJ11* gene*)* and SUR1 (*ABCC8* gene) cause monogenic diabetes by impairing the ability of pancreatic K_ATP_ channels to close in response to metabolically generated ATP or falling Mg-ADP; this leads to hyperpolarized beta cells, which are unable to secrete insulin as blood glucose rises 6, 7. Sulfonylureas can bind and close pancreatic K_ATP_ channels independently of ATP, allowing insulin secretion in both *ABCC8* and *KCNJ11* monogenic diabetes 7, 8. The mechanism by which 6q24 causes TNDM in humans remains unknown; however, small scale human studies suggest first phase insulin secretion may be impaired, supporting treatment with insulin secretagogues (9). A genetic diagnosis is, therefore, crucial in TNDM because affected individuals may be successfully treated with sulfonylureas instead of subcutaneous insulin 10, 11.

Individuals with TNDM remain euglycemic after remission and present later with relapse of diabetes often to an adult diabetologist (10). However, patients are often misdiagnosed because a detailed neonatal history may not be routinely taken. Here, we present 2 cases with TNDM, due to an *ABCC8* mutation and 6q24 imprinting abnormality, who presented as adults when their diabetes relapsed. These cases highlight the importance of TNDM as a crucial etiologic clue that can lead to a monogenic diagnosis and appropriate treatment. Informed consent was obtained from both individuals for the use of their clinical data in this report. Genetic analysis was performed at the Royal Devon and Exeter Molecular Genetics Laboratory in Exeter, United Kingdom.

## Case 1

An asymptomatic 21-year-old Caucasian woman with no history of diabetes was referred by her obstetrician to an adult diabetologist following the birth of her first baby who was macrosomic (birth weight, 5,140 g; birth weight SD score, 3.24); the baby did not have postnatal hypoglycemia. Blood glucose and glycated hemoglobin (A1C) values at different time points are summarized in Table 1.

**Table 1:** Blood glucose and A1C values at different time points relative to 2 pregnancies in case 1

Measure of glycemia	During first pregnancy	After first pregnancy	Before second pregnancy	After second pregnancy
FBG, mmol/L	5.8	7.7	5.4–7.8	8.2
PP-BG, mmol/L	NK	up to 9.0	up to 9.0	6.5–8.9
A1C, %	NK	6.1	6.5	7.2

*A1C,* glycated hemoglobin; *FBG,* fasting blood glucose; *NK,* not known; *PP-BG*, post-prandial blood glucose.

Clinical features at presentation to the diabetes clinic are summarized in Table 2. Initial management comprised dietary changes and regular review. She became pregnant 2 years later and was managed with diet. She gave birth to a healthy baby girl, weighing 3,350 g (body weight SD score, −0.22). On day 6 of life, the baby presented with mild, intermittent hyperglycemia (highest glucose value, 13.1 mmol/L) that did not require glucose-lowering treatment. Genetic analysis revealed that case 1 and her second baby are heterozygous for the *ABCC8* missense mutation, p.Arg1183Trp, previously shown to cause TNDM (12). Postdelivery, case 1 was started on gliclazide 15 mg daily (10% of the standard type 2 diabetes dose) (13) because of rising A1C. Blood glucose monitoring showed fasting hyperglycemia. Her A1C improved from 7.2% (55 mmol/mol) after delivery to 5.9% (41 mmol/mol) at most recent follow up, 6 months after starting gliclazide. She has not reported hypoglycemia, weight gain or other side effects on gliclazide. Capillary glucose testing was not done because she was on oral therapy. The child's diabetes remains in remission needing no treatment, and she is developmentally normal at 4 years of age.

**Table 2:** Clinical features of 2 cases at presentation to the adult diabetes clinic

Clinical features at presentation	Case 1	Case 2
Birth weight, g	2,950	1,600
Birth weight SD score	−1.94	−3.39
Family history of diabetes	Yes, paternal grandmother diagnosed at 45 years of age; tablet treated	No
Clinical examination	Normal	Normal
Acanthosis nigricans	No	No
BMI, kg/m^2^	19.4	22.2
A1C, %	6.1	8.6
A1C, mmol/mol	43	70
GAD antibodies (+/–)	–	–
IA-2A antibodies (+/–)	–	–
UCPCR, nmol/mmol	NK	2.72
C-peptide, nmol/L	0.58	NK

*+,* positive; *–,* negative; *A1C,* glycated hemoglobin; *BMI,* body mass index; *GAD,* glutamic acid decarboxylase; *NK,* not known; *UCPCR*, urine C-peptide creatinine ratio.

## Case 2

A 39-year-old Caucasian woman was referred to a diabetologist with a 3-month history of polyuria, polydipsia, 3 kg weight loss and cystitis. She had developed diabetes soon after birth requiring twice daily insulin for 12 weeks. Her diabetes subsequently remitted, and she had not required treatment until this presentation. Clinical features are summarized in Table 2. Because there was a history of TNDM, genetic analysis was performed, revealing a chromosome 6q24 imprinting abnormality. She started glyburide 2.5 mg/d (25% of the standard type 2 diabetes dose), and her A1C improved to 6.5% (48 mmol/L) after 4 months and 6.7% (50 mmol/L) after 13 months of treatment, with no side effects reported. Her weight increased from 60.5 to 63.8 kg in the first 4 months of treatment, but she lost weight subsequently (63.1 kg after 18 months), and her body mass index remained <25 kg/m^2^ thereafter. Because she was planning a pregnancy when her diabetes relapsed, she was also reassured that the risk of the same type of diabetes in her children would be no higher than that in the general population.

## Discussion

These cases illustrate how a comprehensive history can be fundamental in making a diagnosis of monogenic diabetes in adults, which is crucial for correct clinical management. The mechanism of remission and relapse in TNDM is not known, but many affected individuals can be successfully treated at either stage with oral glucose-lowering drugs, such as sulfonylureas instead of subcutaneous insulin 8, 10, 11, resulting in excellent clinical outcomes 10, 14, 15.

Rafiq et al (11) described successful transfer of 8 of 8 patients with *ABCC8* TNDM from insulin to sulfonylureas, resulting in improved metabolic control in the subsequent 3 months. Regarding long-term efficacy, there is strong evidence of good durability of sulfonylureas over 10 years in *KCNJ11* PNDM (16); such evidence is not present for *ABCC8* neonatal diabetes. However, the similar molecular mechanism and short-term sulfonylurea effectiveness in both subtypes of K_ATP_ channel neonatal diabetes makes it likely that sulfonylurea durability will also be similar.

Although sulfonylureas are the most common treatment for individuals with 6q24 TNDM, there is currently no trial evidence of superiority of these drugs over the other antidiabetic drugs, and long-term sulfonylurea durability remains unknown. In a recent review of 6q24-related TNDM, Garcin et al (10) described successful sulfonylurea treatment in 11 patients (4 before remission and 7 in the relapse phase); however, 4 of 7 patients in the relapse phase also required additional oral therapies, such as metformin and sitagliptin, to maintain metabolic control. This supports the effectiveness of sulfonylureas in 6q24 TNDM to an extent, but further long-term studies in larger cohorts are needed to confirm their durability. Despite this, a genetic diagnosis is still crucial because it can mean avoidance of unnecessary insulin therapy.

In addition to its impact on treatment, identifying a specific genetic etiology also allows accurate calculation of diabetes risk in future offspring and counselling in relation to this. Offspring of people with K_ATP_ channel mutations that cause TNDM are at 50% risk of inheriting the mutation and developing neonatal diabetes and/or adult-onset diabetes. In contrast, this risk with 6q24 TNDM is dependent upon the specific genetic or epigenetic change (17).

Finally, K_ATP_ channel mutations have important implications for clinical management during pregnancy, particularly regarding glyburide therapy (18). Treatment recommendations in mothers with K_ATP_ channel mutations are dependent on the fetal genotype because of differing effects of treatment on fetuses with or without the mutation (18). In the future, more widespread use of cell free fetal DNA analysis in pregnancy (19) will allow noninvasive determination of fetal genotype to assist these treatment decisions.

Despite the importance of diagnosing monogenic diabetes, many patients remain misdiagnosed (20) for several reasons. Firstly, some of the clinical features associated with TNDM are also seen in common polygenic subtypes of diabetes (type 1 and type 2 diabetes), limiting the utility of existing diagnostic tools in the identification of rare monogenic cases. C-peptide and autoantibody testing can assist to an extent, but approximately 8% of those with type 1 diabetes are C-peptide positive (21), and approximately 18% are autoantibody negative (22), making such individuals indistinguishable from those with TNDM mutations based on these biomarkers alone. The Maturity-Onset Diabetes of the Young (MODY) probability calculator may be useful, but it is not validated for patients with TNDM (23). Furthermore, penetrance of some TNDM mutations is variable and not all affected will have a history of neonatal diabetes 24, 25. Low birth weight may point towards the presence of monogenic diabetes under such circumstances (25); however, this feature is not specific to TNDM.

Despite these caveats, the presence of neonatal diabetes is one of the strongest clues for an underlying monogenic etiology because >82% of individuals with neonatal diabetes have a monogenic cause (4). International guidelines recommend genetic testing for all patients with neonatal diabetes irrespective of clinical features (26). Adoption of these guidelines is improving, particularly in pediatrics, and with better case ascertainment and appropriate genetic counselling, misdiagnosis should become less frequent in the future. However, large numbers of cases currently remain unascertained, and it is therefore crucial to remain vigilant for clues pointing towards a monogenic etiology in adults.

## Conclusions

Adult diabetologists should routinely ask patients about a personal or family history of neonatal diabetes and low birth weight. The presence of diabetes under 6 months of age makes a monogenic etiology very likely, and should prompt clinicians to undertake genetic testing. In many cases, a genetic diagnosis will result in optimization of treatment, accurate genetic counselling and improved management of pregnancy.

## Supplementary Material

Data Profile
